# The chromosome-level genomes of the herbal magnoliids *Warburgia ugandensis* and *Saururus chinensis*

**DOI:** 10.1038/s41597-024-03229-9

**Published:** 2024-05-30

**Authors:** Liuming Luo, Dongming Fang, Fang Wang, Qiongqiong Lin, Sunil Kumar Sahu, Yali Song, Jingmin Kang, Xuanmin Guang, Min Liu, Shixiao Luo, Gang Hao, Huan Liu, Xing Guo

**Affiliations:** 1 College of Life Science, South China Agricultural University, Guangzhou, 510642 China; 2 State Key Laboratory of Agricultural Genomics, Key Laboratory of Genomics, Ministry of Agriculture, BGI Research, Shenzhen, 518083 China; 3 College of Life Sciences, University of Chinese Academy of Sciences, Beijing, 100049 China; 4 BGI Research, Wuhan, 430074 China; 5 BGI Research, Beijing, 102601 China; 6 Key Laboratory of Plant Resources Conservation and Sustainable Utilization, South China Botanical Garden, Chinese Academy of Sciences, Guangzhou, Guangdong 510650 China; 7 South China National Botanical Garden, Guangzhou, 510650 China

**Keywords:** Plant evolution, Comparative genomics, Bioinformatics

## Abstract

*Warburgia ugandensis* and *Saururus chinensis* are two of the most important medicinal plants in magnoliids and are widely utilized in traditional Kenya and Chinese medicine, respectively. The absence of higher-quality reference genomes has hindered research on the medicinal compound biosynthesis mechanisms of these plants. We report the chromosome-level genome assemblies of *W. ugandensis* and *S. chinensis*, and generated 1.13 Gb and 0.53 Gb genomes from 74 and 27 scaffolds, respectively, using BGI-DIPSEQ, Nanopore, and Hi-C sequencing. The scaffold N50 lengths were 82.97 Mb and 48.53 Mb, and the assemblies were anchored to 14 and 11 chromosomes of *W. ugandensis* and *S. chinensis*, respectively. In total, 24,739 and 20,561 genes were annotated, and 98.5% and 98% of the BUSCO genes were fully represented, respectively. The chromosome-level genomes of *W. ugandensis* and *S. chinensis* will be valuable resources for understanding the genetics of these medicinal plants, studying the evolution of magnoliids and angiosperms and conserving plant genetic resources.

## Background & Summary

Among angiosperms, Amborellales, Nymphaeales and Austrobaileyales (collectively referred to as ANA grade) are followed by the rapid diversification of the remaining angiosperms or mesangiosperms^1,2^. The major mesangiosperm lineages are the eudicot, monocot and magnoliid clades^3^. Among the magnoliid clades, there are four orders, namely Canellales, Piperales, Magnoliales and Laurales. Long read sequencing has enabled chromosome-level assembly of many important plant genomes^4–6^. Although the number of sequenced magnoliid genomes has been increasing recently^3,7–14^, no Canellales genomes have been published. Similarly, only a few Piperales genomes have been published. In addition, there are many unanswered questions about the early diversification of mesangiosperm and the molecular mechanisms that have contributed to diversification and evolution within lineages^15–22^. *Warburgia ugandensis* is a medicinal plant belonging to Canellales, magnoliid.While, *Saururus chinensis* is a Piperales medicinal plant, athough a genome version of *S. chinensis* has been released^23^, deciphering their genomes will provide valuable and complementary genomic resources for future investigations into systematic evolution and medicinal components in the magnoliids.

*W. ugandensis*, a member of the Canellaceae, Canellales, is widely used for its pharmacological properties. The medicinal effectiveness of *W. ugandensis* is mainly associated with abundant terpenoids, particularly drimane and coloratane-type sesquiterpenoids, as well as fatty acid derivatives in the leaf and bark tissues^24–26^. The ever-increasing global demand for *W. ugandensis* and its use in treating and managing various diseases has led to overexploitation of this species, coupled with the difficulty of introducing it into temperate and cold regions, resulting in a drastic decrease in its population size^15,27^. *W. ugandensis* is therefore listed as a vulnerable species by the International Union for Conservation of Nature and Natural Resources (IUCN)^28^. Conservation strategies and rapid propagation techniques need to be implemented to protect this “miraculous species” from extinction. However, studies on the molecular characterization and biosynthesis of terpenoids and unsaturated fatty acids in *W. ugandensis* are relatively limited. Little is known about its genetic background, and its karyotype has not been previously reported. Therefore, genome sequencing is crucial for understanding the genetic background of this species, and can in turn lay a solid foundation for the development of its medicinal value and species conservation.

*S. chinensi*s, with a chromosome number of 22 (2n = 2x = 22), is a perennial aquatic herb belonging to the Saururaceae, Piperales, and has been listed in the 2020 edition of Pharmacopoeia of the People’s Republic of China^29^. It is not only used as an ornamental aquatic plant but also has a long history of traditional medicinal use in China. It has significant analgesic, hypoglycemic, hepatoprotective, anti-angiogenesis, antioxidant, and anti-inflammatory^30–32^ properties. A comprehensive review of the taxonomic classification, morphology, and geographical distribution of Saururaceae plants revealed that Saururaceae is an early-branching family of Piperales and a stable component of ancient herbaceous plants^33^. However, despite the publication of a version of the *S. chinensis* genome by previous researchers, further research on the pharmacological components of *S. chinensis* requires a higher quality genome and additional transcriptome data from different tissues as a research foundation. Overall, genomics-based approaches for *S. chinensis* can provide valuable insights not only into the origin and early evolution of flowering plants (angiosperms), but also into how to directly and significantly modify one or more genes in the genomes of medicinal species, or to identify effective genetic markers and genes for molecular breeding^23^.

In the present study, we constructed high-quality genome assemblies for *W. ugandensis* and *S. chinensis* using the integration strategy of short reads (BGI-DIPSEQ sequencing), long reads (nanopore sequencing) and Hi-C reads (proximity ligation chromatin conformation capture). The final assembled genomes were 1.13 Gb and 533.01 Mb in length with scaffold N50 values of 82.97 Mb and 48.53 Mb for *W. ugandensis* and *S. chinensis*, respectively. A total of 1.12 Gb (99.49%) and 531.46 Mb (99.59%) of assembled genome sequences were successfully anchored on 14 and 11 chromosomes, respectively (Table 1). A total of 24,739 protein-coding genes were predicted for *W. ugandensis*, and 20,561 protein-coding genes for *S. chinensis* (Table 2). The percentage of functionally annotated genes in the *W. ugandensis* and *S. chinensis* accounted for as high as 99.94% and 99.93%, respectively (Table 2).

**Table 1:** Genome assembly and assessment of *W. ugandensis* and *S. chinensis*.

Assembly		*W. ugandensis*	*S. chinensis*
Genome-sequencing depth (X)	Nanopore sequencing	117.72	123.42
	BGI-DIPSEQ sequencing	132.43	260.67
	Hi-C	258.49	187.16
Estimated genome size by *k*-mer (Gb)		1.16	0.56
Total scaffolded assembly size (Mb); Total scaffolds		1129.60; 74	533.64; 27
Scaffolds N50 (Mb)		82.97	48.53
Contigs N50 (Mb)		21.63	14.96
Longest contig (Mb)		59.14	40.04
GC content (%)		36.41	37.76
Completeness BUSCOs (%)		98.50	98.00
Largest scaffold (Mb)		106.07	68.54
Gaps, combined length (kb)		62.5	30.7
N50 in kb of scaffolds; Count > N50 length		82,969.85; 7	48,534.73; 5
Chromosome number (%)		14	11
Anchor ratio (%)		99.49	99.59

**Table 2:** Genome annotation of *W. ugandensis* and *S. chinensis*.

Annotation	*W. ugandensis*	*S. chinensis*
Number of predicted protein-coding genes	24,739	20,561
Average gene length (bp)	1255.24	933.63
Average exon length (bp)	217.12	220.48
Average exon number per gene	5.78	4.23
Average intron length (bp)	1336.33	902.07
Percentage of repeat sequence (%)	54.83	54.81
LTR/Copia (%)	5.09	5.82
LTR/Gypsy (%)	34.48	23.66
LINE (%)	8.46	3.48
SINE (%)	0.63	0.01
DNA transposons (%)	3.85	8.35
Percentage of functional annotation genes	99.94%	99.93%

## Methods

### Sample preparation, DNA/RNA extraction, library construction and sequencing

Samples of *W. ugandensis* were collected from plants grown in the greenhouse of Wuhan Botanical Garden, and *S. chinensis* was collected from the lakeside of South China National Botanical Garden. For second-generation short-read library construction and sequencing, DNA was extracted using the CTAB (cetyltrimethylammonium bromide) method^34^ on fresh young leaves. The library was sequenced on the BGI-DIPSEQ platform^35^, generating ~149 Gb and ~137 Gb of 100 bp paired-end reads with an insert size of ~250 bp for *W. ugandensis* and *S. chinensis*, respectively. For subsequent analyses, such as genome size estimation and ONT assembly polishing, only high-quality reads were used.

For ONT library construction and sequencing^36^, after grinding fresh young leaf tissues of *W. ugandensis* and *S. chinensis* in liquid nitrogen, extraction was performed. With the LSK108 kit (SQK-LSK108, Oxford), we generated the library and performed sequencing on the Nanopore GridION X5 sequencer using five flow cells. The base calling was performed using Guppy (version 4.0.11) in the MinKNOW package. There were ~132 Gb (118x) and 65 Gb (123x) of raw data for *W. ugandensis* and *S. chinensis*, respectively, in total available for assembly (Table 1).

We collected fresh young leaves, mature leaves, stems close to the apical meristem, stems far from the apical meristem, rhizomes, root tissues, budding flowers, full-blooming flowers and flowers nearing the senescence stage of *S. chinensis* and the fresh young leaves of *W. ugandensis* for transcriptome sequencing, three biological replicates for each sample. Total RNA was extracted using the TIANGEN Kit with DNase I and then processed using the NEBNextUltra^TM^ RNA Library Prep Kit to create a pair-end library with a 250 bp insert size. The RNA libraries were subsequently sequenced on the BGI-DIPSEQ platform. After the filtering of low-quality data by the Trimmomatic (version 0.39)^37^ with the parameters: ILLUMINACLIP:adapter.fa:2:30:10 LEADING:5 TRAILING:5, 6 Gb of 100 bp paired-end data for each tissue was used for later analysis.

### Hi-C library construction and sequencing

The construction of Hi-C libraries was performed by utilizing the DpnII restriction enzyme and following the method developed by BGI QingDao Institute^38^. The chromatin digested with DpnII was labeled at the ends with biotin-14-dATP (Thermo Fisher Scientific, Waltham, MA, USA). Subsequently, the DNA was extracted, purified, and sheared using Covaris S2 (Covaris, Woburn, MA, USA). Hi-C libraries were subjected to sequencing on a BGI-DIPSEQ platform, generating ~290 Gb (258x) and ~100 Gb (187x) of data with 100 bp paired-end reads (Table 1). Hi-C data enabled the identification of 14 chromosomes for *W. ugandensis* and 11 for *S. chinensis*, which was consistent with the reported chromosome numbers of *S. chinensis*^23^.

### Genome size, heterozygosity and ploidy level evaluation

**Fig. 1:**
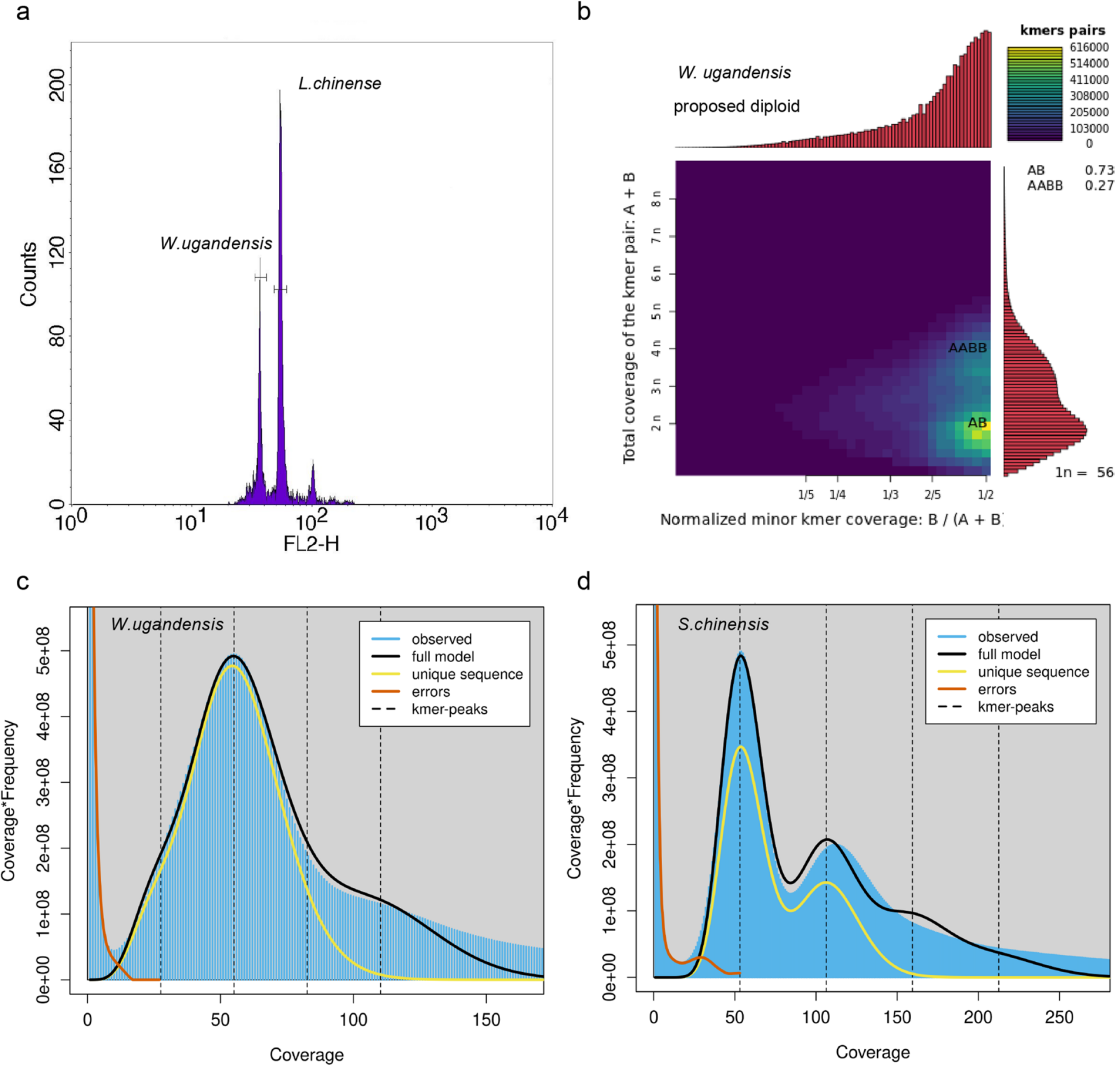
Genome sizes and ploidy levels estimated by flow cytometry experiment, smugeplot and survey analyses. (**a**) Flow cytometry experiment of *W. ugandensis* conducted with *Liriodendron chinense* as the reference (**b**) Ploidy assessment of *W. ugandensis* using a Smugeplot. (**c,d**) Genome survey based on *k*-mer (*k* = 17) analysis of *W. ugandensis* and *S. chinensis*, respectively.

Two approaches were used to estimate the size of *W. ugandensis* genome: flow cytometry and *k*-mer spectral analysis of 60x BGI-DIPSEQ short reads. The flow cytometry technique was conducted with *Liriodendron* as the reference, generating 1.16 Gb of *W. ugandensis* (Fig. 1a). Additionally, we used the *k*-mer frequencies with the size of 17 to assess the genome size from short BGI-DIPSEQ reads. According to the results of 17-mer frequency distribution analysis with GenomeScope 2^39^, a 1.16 Gb genome size of *W. ugandensis* was estimated (Fig. 1c and Table 1). While *k*-mer analysis estimated the *S. chinensis* genome size to be 555 Mb (Fig. 1d and Table 1), which is similar in size to 553 Mb obtained through flow cytometry analysis by Xue *et al*.^23^. To minimize the sequencing error rate, strict quality control was performed using SOAPfilter (version 2.2)^40^. To estimate the heterozygosity of the genomes, we used the Genome Analysis Toolkit (GATK) 4.2.3.0 for variant-calling of whole-genome short-read data, resulting in the heterozygosity values of 0.24% and 2.50% for *W. ugandensis* and *S. chinensis*, respectively. Given that the ploidy of *W. ugandensis* is unclear, we used Smudgeplot (https://github.com/KamilSJaron/smudgeplot) to estimate its ploidy and found that it may be diploid (Fig. 1b).

### Genome assembly and assessment of the assembly quality

The raw long reads obtained from ONT were used for *de novo* assembly using the NextDenovo assembler (version 2.2, https://github.com/Nextomics/NextDenovo) with the parameters: read_ cutoff = 1 k, seed_cutoff = 26,766 (*W. ugandensis*) and 16,118 (*S. chinensis*). The NextPolish (version 1.3.0, https://github.com/Nextomics/NextPolish)^41^ was subsequently applied to polish the initial draft assembled contigs with six rounds (two rounds with ONT long reads and four rounds with short reads). Purge dups (version 1.2.3)^42^ was then used to select contigs of *S. chinensis* to retain for the haploid assembly by taking into account mapped read coverage using short read and Minimap2 alignments^43^.

**Fig. 2:**
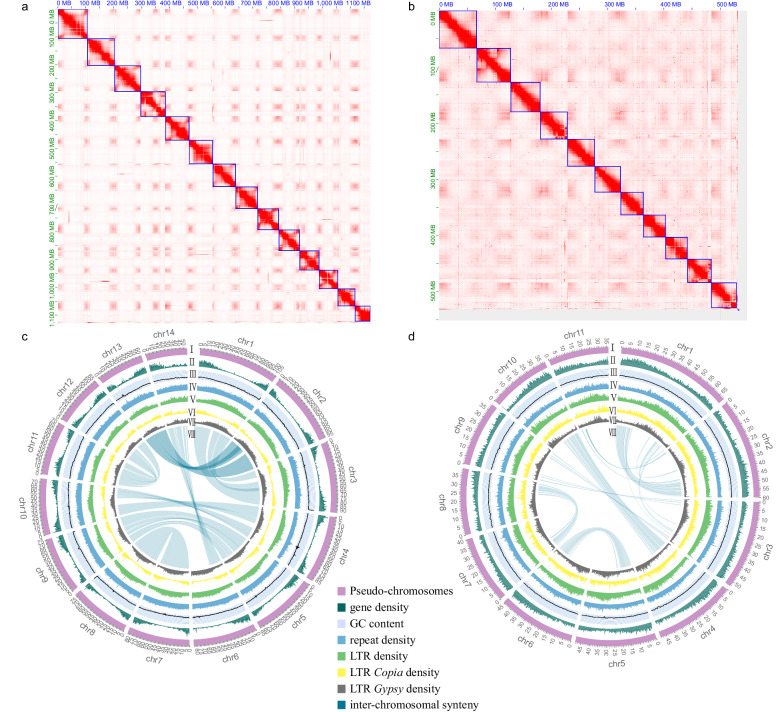
Overview of the chromosomal features of two magnoliid genomes. (**a,b**) Hi-C interaction heatmaps of 14 chromosomes of *W. ugandensis* and 11 chromosomes of *S. chinensis*. (**c,d**) Circos plots of *W. ugandensis* and *S. chinensis*. The concentric circles from the outermost to the innermost regions show the chromosome and megabase values, (I) Pseudo-chromosomes, (II) gene density, (III) GC content, (IV) repeat density, (V) LTR density, (VI) LTR *Copia* density, (VII) LTR *Gypsy* density and (VIII) inter-chromosomal synteny (calculated in non-overlapping 200 Kb sliding windows).

Hi-C paired-end reads were trimmed to remove low-quality bases and adapter sequences using Trimmomatic (version 0.39)^37^. To calculate the contact frequency, all the filtered reads were aligned to contig assembly using Juicer (https://github.com/aidenlab/juicer, version 3)^44^. Then, 3D-DNA (version 180922)^45^ pipeline was subsequently run with two iterative rounds for misjoining correction (-r2), and other parameters were set to the default values. Manual checking and refinement of the draft assembly were carried out with Juicebox assembly tools (version 1.11.08)^46^ (Fig. 2a,b).

The assembly evaluations for the genomes are provided as follows: First, mapping of the 1,614 conserved core eukaryotic genes from the BUSCO dataset (embryophyta_odb10, BUSCO v5)^47^, resulted in 98.5% and 98.0% (Table 1) of the core eukaryote genes recovered for the majority of the *W. ugandensis* and *S. chinensis* genome assemblies, respectively. We then mapped the RNA reads to the draft assemblies to evaluate the RNA reads mapping rate using Hisat2^48^, with the mapping rate > 93%. Taken together, these results indicated good genome assembly qualities for this sequenced species.

In this study, a comparison of the genome of *S. chinensis* with a previously published genome revealed a similar genome size, but the current study identified a smaller number of gaps in the genome (132 gaps, size: 30,702 bp) compared to the previous version (804 gaps, size: 80,400 bp). Additionally, this study presented a lower number of contigs (75 contigs) with a higher contig N50 (14.96 Mb) compared to the previous version (842 contigs, Contig N50: 1.40 Mb). Moreover, the fragmented BUSCOs (F) and missing BUSCOs (M) in the genome were reduced in this study (BUSCO (F + M): 2.89) compared to the previous version (BUSCO (F + M): 5.76). The assembled genome of *S. chinensis* in our study is of high quality, potentially due to the large amount and deep depth of the short reads, long reads and Hi-C reads used by us. In addition, we used additional tissues and libraries for the transcriptome.

### Repetitive element annotation

Repeat sequences in the genomes were identified using a combination of *de novo* and homology-based approaches. For *de novo* approaches, we used LTR_retriever^49^, LTR_FINDER (version 1.0.7)^50^, and RepeatModeler2^51^ to construct a new repeat library and later RepeatMasker (version 4.0.6)^52^ was used to annotate the repeat elements. Finally, tandem repeats were searched across the genome using the software Tandem Repeats Finder (version 4.07)^53^. For homology-based approaches, repeat elements were predicted by employing a combination of homology-based comparisons in RepeatMasker (version 4.0.5) and RepeatProteinMask^52^. Both *W. ugandensis* and *S. chinensis* displayed moderate quantity repetitive elements, which accounted for 54.83% and 54.81% of assemblies, respectively (Fig. 2c,d and Table 2), while the percentage of *Copia* elements was 5.09% and 5.82%, respectively (Table 2).

### Protein-coding gene prediction

**Fig. 3:**
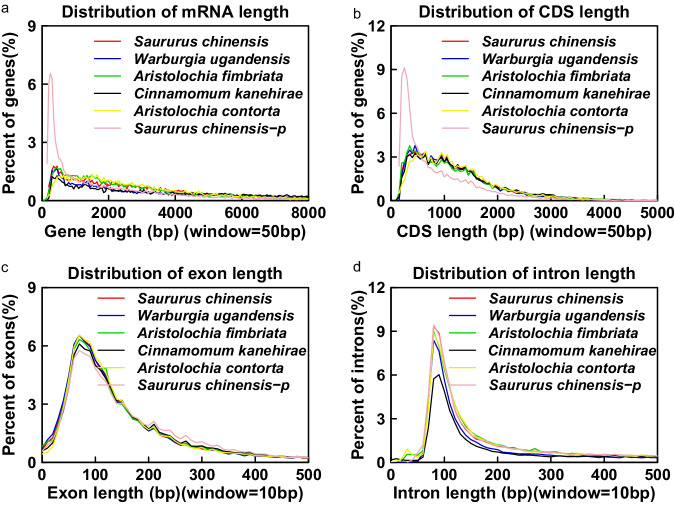
Comparison of the distribution of gene elements for each gene among the six magnoliid species. (**a**) mRNA length. (**b**) CDS length. (**c**) Exon length. (**d**) Intron length. The x-axis represents the length and the y-axis represents the density of genes. *Saururus Chinensis-p* refers to the genome published previously.

The prediction of protein-coding gene sets was inferred using *de novo* gene prediction, homology-based annotation and evidence-based gene prediction. In the *De novo* approach, gene prediction was performed on a repeat-masked genome using Augustus (version 3.0.3)^54^, GlimmerHMM (version 3.0.1)^55^, and SNAP (version 11/29/2013)^56^. We analyzed the repeat-masked genomes of *W. ugandensis* and *S. chinensis* to predict coding genes, respectively. In the homology comparisons, homologous gene prediction was achieved by comparing the amino acid sequences of *Amborella trichopoda*, *Arabidopsis thaliana*, *Oryza sativa*, four related species (*Aristolochia fimbriata*, *Chimonanthus salicifolius*, *Liriodendron chinense*, and *Litsea cubeba*) using GeMoMa (version 1.3.1)^57^, and uniprot database (release 2021_04). By comparing with protein sequences covering the complete genome, TBLASTN (version 2.2.18) (e-value cutoff: 1e-5)^58^ was used to predict putative homologous genes. Then, GeneWise (version 2.2.0)^59^ was utilized to process the alignment regions and obtain precise exon and intron information. In the RNA-seq-based prediction approach, gene prediction was carried out by aligning the clean RNA-seq reads generated in this study against the assembled genomes using Hisat2 (version 2.0.4)^48^. cDNAs were identified through a genome-guided approach using StringTie (version 1.2.2)^60^ and then mapped back to the genome using PASA (version 2.3.3)^61^. The resulting cDNA sequences assembly from Trinity (version 2.6.6)^62^ were aligned to the *W. ugandensis* and *S. Chinensis* genome sequences using BLAT (version 34 × 12)^63^, respectively. After predicting genes using the aforementioned three methods, a non-redundant gene set was generated through BRAKER2^64^ pipeline. A total of 24,739 and 20,561 protein-coding genes were predicted in *W. ugandensis* and *S. chinensis*, individually. As shown in Fig. 3, we can also observe that the mRNA length distribution, CDS length distribution, intron length, and exon number of *W. ugandensis* and *S. chinensis* in this study are similar to the distribution characteristics of other Magnoliid species genomes (*Aristolochia fimbriata*^65^, *Cinnamomum kanehirae*^66^, *Aristolochia contorta*^67^) published previously. However, the mRNA length distribution and CDS length distribution of the previously published *S. chinensis* genome are significantly higher than the percentage of other close related species in magnoliids, including the *S. chinensis* genome from this study. Moreover, the fragmented BUSCOs (F) and missing BUSCOs (M) in the gene set were reduced in this study (BUSCO (F + M): 4.77) compared to the previous version (BUSCO (F + M): 8.98). Our statistics estimated that we assembled a high-quality genome for *S. chinensis* (Table 1, Fig. 3).

### Functional annotation

The protein-coding genes were subjected to functional annotation by performing sequences against similarity and domain conservation. Initially, a homolog search against public protein databases was conducted using BLASTP (e-value cutoff: 1e-5) to identify protein-coding genes with the following filtering criteria: -tophit 5, amino acid identify > 0.3, and match length cutoff > 0.5. The following public protein databases were used: SwissProt (release-2020_05)^68^, KEGG (59.3)^69^, TrEMBL (release-2020_05)^68^ and NCBI non-redundant protein NR database (20201015). Subsequently, InterProScan (version 5.28-67.0)^70^ was used to provide functional annotation by detecting and classifying domains and motifs. Finally, the annotation rates for *W. ugandensis* and *S. chinensis* were found to be 99.94% and 99.93% respectively (Table 3).

**Table 3:** Statistics of gene functional annotations of *W. ugandensis* and *S. chinensis*.

	*W. ugandensis*		*S. chinensis*	
	Number	Percentage	Number	Percentage
Total	24,739	100%	20,561	100%
Nr	24,595	99.42%	20,370	99.07%
Swissprot	21,253	85.91%	17,586	85.53%
KEGG	19,452	78.63%	16,567	80.57%
KOG	19,671	79.51%	16,504	80.27%
TrEMB	24,665	99.70%	20,505	99.73%
Interpro	24,458	98.86%	20,281	98.64%
GO	16,857	68.14%	13,911	67.66%
Overall	24,723	99.94%	20,546	99.93%

Note: The “Number” column in “Overall” refers to the union of protein-coding genes annotated by the seven public protein databases used for functional annotation. The “Percentage” column in “Overall” indicates the proportion of all protein-coding genes obtained from functional annotation.

### Phylogenetic analyses

**Fig. 4:**
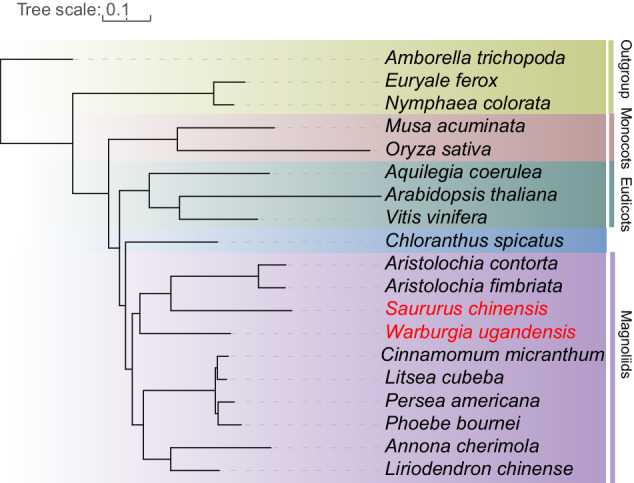
Phylogenetic tree among 19 of representative species. A phylogenetic tree among 17 representative species, combining the two Magnoliid genomes in this study, was constructed using the maximum likelihood method.

The protein-coding genes of 17 of representative species combining the two Magnoliid genomes were selected for gene families analysis using OrthoFinder (version 2.3.14)^71^ with default parameters, among which *A. trichopoda* was set as an outgroup. Totally, 601 low-copy Orthogroups were used for phylogenetic tree construction. The protein sequences from the 601 low-copy Orthogroups were extracted and aligned by using MAFFT (version 7.310)^72^. The aligned sequences were concatenated into a super matrix and subsequently input into IQ-TREE (version 1.6.1)^73^ with “-bb2000-alrt 1000” to construct phylogenetic tree. The topology revealed a robust topology and supported sister relationship between magnoliids and Chloranthus (bootstrap support = 100), which together formed a sister group relationship (bootstrap support = 100) with eudicots and monocots (Fig. 4b). Magnoliid comprises four orders and no genome was available for any species of the Canellales order. What sets our research apart from previous angiosperm phylogenetic trees is that it is the first time we have combined the genomes of all four orders of Magnoliid with those of monocotyledonous plants, dicotyledonous plants, and outgroups to construct a comprehensive phylogenetic tree. This approach enhances the persuasiveness of our results.

## Data Records

The Nanopore, Hi-C, BGI-DIPSEQ and RNA sequencing data that were used for the genome assembly and annotation have been deposited in the Genome Sequence Archive in National Genomics Data Center (NGDC) Genome Sequence Archive (GSA) database with the accession number CRA014162^74^ under the BioProject accession number PRJCA022413^75^. All the genomic sequencing raw data were also deposited in the CNGB Nucleotide Sequence Archive (CNSA) under accession numbers CNP0004586^76^ and CNP0003309^77^ for *W. ugandensis* and *S. chinensis*, respectively. The final contigs and chromosome assemblies are submitted to the NCBI under the accession number GCA_035236585.1^78^, GCA_035235625.1^79^ of *W. ugandensis* and *S. chinensis*, respectively. The contigs and chromosome-scale genome assemblies were also deposited in the Genome Sequence Archive^80^ in the National Genomics Data Center^81^ (CNCB/NGDC) under the BioProject accession PRJCA018454, with accession numbers GWHDQZE00000000^82^ and GWHDQZF00000000^83^ for *W. ugandensis* and *S. chinensis*, respectively. The annotation files are available in figshare^84^. All the other data generated or analyzed during this study are included in this article.

## Technical Validation

Completeness assessment was performed using BUSCO (Bench-marking Universal Single-Copy Orthologs) version 3.0.1^47^ with the Embryophyta odb10 database. Among the 1,614 core Embryophyta genes, 98.50% and 98.00% were identified in the *W. ugandensis* and *S. chinensis*, respectively (Table 1). To further evaluate the completeness of the assembled genome, we performed short-read mapping using clean raw data. In total, 99.64% and 96.33% of them were properly paired with *W. ugandensis* and *S. chinensis*, respectively. The transcriptome sequences were assembled by using Bridger tool^85^, then mapping to the scaffold assembly was performed by using the BLAT software^63^, 93.32% and 99.64% of them were paired with *W. ugandensis* and *S. chinensis*, respectively on average. The BUSCO analysis was again performed after the Hi-C assembly which gave similar results as those of the ONT genome assemblies.

### Supplementary information

Supplementary Information

## Data Availability

No specific script was used in this work. The codes and pipelines used in data processing were all executed according to the manual and protocols of the corresponding bioinformatic tools (Table S1). The software versions are described in the Methods section.
